# Quinolones for the Treatment of *Neisseria Gonorrhoeae* and *Chlamydia Trachomatis*

**DOI:** 10.1155/S1064744993000250

**Published:** 1993

**Authors:** Sebastian Faro

**Affiliations:** Department of Gynecology and Obstetrics The University of Kansas School of Medicine 3901 Rainbow Blvd. Kansas City KS 66160-7316 USA

## Abstract

The most commonly sexually transmitted bacteria are *Neisseria gonorrhoeae* and *Chlamydia trachomatis.* The quinolones ofloxacin and ciprofloxacin have been shown to have activity against both of these bacteria in vitro and in vivo. Ofloxacin is particularly well suited for the treatment of *N. gonorrhoeae* and *C. trachomatis* cervical infection, which can be considered the earliest manifestation of pelvic inflammatory disease (PID). Not only can ofloxacin be effectively used as a single agent, it is also useful in treating urinary tract infections caused by Enterobacteriaceae. Although it has moderate activity against anaerobes in general, ofloxacin does have activity against the anaerobes commonly isolated from female patients with soft tissue pelvic infections. Thus, ofloxacin has the potential for being utilized to treat early salpingitis.


Infectious Diseases in Obstetrics and Gynecology 1:108–113 (1993)
© 1993 Wiley-Liss, Inc.

### Quinolones for the Treatment of *Neisseria gonorrhoeae* and *Chlamydia trachomatis*

**Sebastian Faro**

*Department of Gynecology and Obstetrics, The University of Kansas School of Medicine, Kansas City, KS*

### Abstract

**The most commonly sexually transmitted bacteria are *Neisseria gonorrhoeae* and *Chlamydia trachomatis*. The quinolones ofloxacin and ciprofloxacin have been shown to have activity against both of these bacteria in vitro and in vivo. Ofloxacin is particularly well suited for the treatment of *N. gonorrhoeae* and *C. trachomatis* cervical infection, which can be considered the earliest manifestation of pelvic inflammatory disease (PID). Not only can ofloxacin be effectively used as a single agent, it is also useful in treating urinary tract infections caused by Enterobacteriaceae. Although it has moderate activity against anaerobes in general, ofloxacin does have activity against the anaerobes commonly isolated from female patients with soft tissue pelvic infections. Thus, ofloxacin has the potential for being utilized to treat early salpingitis.** © 1993 Wiley-Liss, Inc.

Key words
Ofloxacin, bacterial infections, sexually transmitted diseases

*Neisseria gonorrhoeae* and *Chlamydia trachomatis* are the two most common sexually transmitted bacteria. They are responsible for the majority of diseases involving the female reproductive tract that can result in devastating sequelae. The spectrum of diseases secondary to infection with either or both of these bacteria encompasses relatively simple infections such as urethritis and cervicitis and more complex infections such as Bartholin's gland and Skene's gland abscesses, endometritis, salpingitis, disseminated gonococcal disease, and gonococcal arthritis.[1–6] Infection of the upper genital tract can and often does result in infertility. Even if the patient is able to achieve pregnancy, she is at considerable risk for the development of an ectopic pregnancy.[7–10] In addition, an individual who has had salpingitis is often left with pelvic adhesions and chronic pelvic pain or develops a pyosalpinx that can result in a hydrosalpinx or tubo-ovarian abscess.[7,11–13] Such an individual must often undergo a hysterectomy with removal of her ovaries, requiring subsequent hormonal replacement. Therefore, the best defense against the patient's developing advanced disease is early recognition of the initial infection along with appropriate treatment and follow-up management.

Early gonococcal infection, i.e., urethritis and cervicitis, is currently treated with ceftriaxone (Rocephin, Roche Laboratories, Nutley, NJ), 150 mg IM, followed by doxycycline, 100 mg orally twice daily for 7 days, to eradicate any chlamydial infection. This treatment regimen, recommended by the Centers for Disease Control (CDC),[14] is a logical one because these two bacteria commonly coinfect individuals. Of women with gonococcal cervicitis, approximately 26% will be coinfected with *C. trachomatis*.[15] Since most patients are treated prior to receipt of the culture results, it is important that antibiotic therapy be effective against both *N. gonorrhoeae* (including penicillinase-pro-

**Address correspondence/reprint requests to Dr. Sebastian Faro, Department of Gynecology and Obstetrics, The University of Kansas School of Medicine, 3901 Rainbow Blvd., Kansas City, KS 66160-7316.**

Received August 18, 1993
Accepted August 19, 1993

Review Article

**TABLE 1. Risk factors for sexually transmitted disease**

| |
|---|
| 15 to 25 years of age (age group most vulnerable) |
| Use of oral contraceptives |
| Presence of an IUD |
| Multiple sex partners |
| Exposure to an individual with an STD |
| Presence of an STD |
| History of an STD |
| History of PID |

ducing strains) and *C. trachomatis*. In addition, patients seen in sexually transmitted disease (STD) clinics and emergency rooms are not usually compliant in their treatment regimen. If they are not treated at the time of their initial examination but are asked to return for the administration of appropriate treatment when the laboratory data are known, they are not likely to return. On the other hand, the administration of two antibiotics, one injectable and one oral, is costly and time consuming. Therefore, an effective single antibiotic agent for the treatment of both infections would be less costly, more effective, and more convenient.

### PATIENT EVALUATION

Appropriate treatment of the patient with an STD begins with an understanding of the profile of the individual at risk. The first indication that an individual may be harboring *N. gonorrhoeae* or *C. trachomatis* is the presence of another sexually transmitted organism such as *Trichomonas vaginalis*, herpes virus, or the human papillomavirus. Demographic characteristics of patients at risk for acquiring an STD are given in Table 1. A detailed sexual history is important in treating the sexually active patient. Germane to the treatment and follow-up of such a patient is determining whether specimens for the culture of *N. gonorrhoeae* and *C. trachomatis* should also be obtained from the oropharynx and rectum. The clinician should also ascertain whether the patient or her sexual partner has multiple partners. Questions should be asked with regard to the presence of genital lesions, past or present, and the existence of lower abdominal pain, no matter how mild or vague. The patient should be further queried regarding the use of OCPs and any abnormal vaginal bleeding, i.e., irregular menstrual bleeding, breakthrough bleeding, or postcoital bleeding.

**TABLE 2. Ulcerative lesions of the genital tract**

| |
|---|
| Syphilis |
| Chancroid |
| Lymphogranuloma venereum |
| Granuloma inguinale |
| Herpes |

The evaluation begins with a physical examination, reserving the pelvic assessment until last. The pelvic examination should include a thorough assessment of the external genitalia with retraction of the clitoral hood and inspection between the crural folds. A colposcopic examination will facilitate the detection of vulvar lesions. The labia should be thoroughly painted with 5% acetic acid to highlight abnormal tissue and discern acetowhite epithelium. If an ulcerative lesion is found, it should be evaluated for the STDs listed in Table 2.

Ulcerative lesions may or may not be differentiated on physical examination. The chancre of syphilis is typically a clean-appearing, painless lesion, whereas the lesions of the other bacterial STDs are painful, with ragged borders, and have an overall "dirty" appearance. Herpetic ulcers vary in size from pinpoint lesions to large, crater-like ulcers and are typically very painful. Appropriate specimens should be obtained for Gram stain, Giemsa stain, dark-field examination, and culture. The laboratory should be consulted for the precise method for obtaining, transporting, and labeling the specimens. In this way, the specimens will be processed appropriately for retrieval of the desired organisms.

The vestibule of the vagina should be inspected for the presence of a purulent discharge. The urethra and Skene's and Bartholin's glands should be palpated to determine if a purulent exudate is present. If an exudate is present, a specimen should be obtained for Gram stain and culture for the isolation of *N. gonorrhoeae*, *C. trachomatis*, *Mycoplasma hominis*, and *Ureaplasma urealyticum*. Again, the entire genitalia should be examined for ulcerative or abnormal growths. A biopsy specimen should be taken of any lesion that is suspicious for malignancy.

The vagina should be inspected for the characteristics of any discharge: color, consistency, pH, and odor. A healthy vaginal discharge has a pH of 3.8 to 4.2. A pH value between 4.2 and 4.5 may indicate an abnormality, whereas a pH > 4.5 definitely indicates an abnormal vaginal microflora. Lower hydrogen ion concentrations (pH > 4.5) indicate the presence of high counts of anaerobic bacteria or *T. vaginalis*.

The evaluation of the cervix begins with an assessment of the endocervical tissue: is it hypertrophic, erythematous, necrotic, or ulcerated? Is an endocervical mucopurulent discharge present? It should also be noted whether the cervix bleeds briskly when gently touched with a cotton-tipped applicator. The aforementioned characteristics are all indications that an infection may be present. An endocervical specimen should be obtained with a Dacron-tipped applicator with a plastic or metal shaft. Cultures should be obtained for *C. trachomatis* and *N. gonorrhoeae*. A Gram stain of the endocervical discharge is helpful. If the specimen does not reveal the presence of squamous epithelial cells, *Candida albicans*, or *T. vaginalis*, it can be considered a valid specimen not containing a vaginal discharge. WBCs with gram-negative diplococci may indicate *N. gonorrhoeae*. However, if there are WBCs but no gram-stainable bacteria, a tentative diagnosis of *C. trachomatis* infection can be made, although an *M. hominis* or *U. urealyticum* infection cannot be excluded. The Gram stain should not be performed in lieu of obtaining specimens for the isolation of *N. gonorrhoeae, C. trachomatis*, or other indicated STDs. A pap smear should be obtained if several months have elapsed since the patient's last pap smear or if she has evidence of cervicitis. The pap smear will help to establish the presence of an inflammatory condition or, if all culture data are negative, a viral infection. In addition to cultures for *N. gonorrhoeae* and *C. trachomatis*, specimens should be obtained for *M. hominis* and *U. urealyticum*.

Next, a bimanual examination should be performed. Although the patient may be suspected of having an uncomplicated lower genital tract infection, it is crucial to the preservation of her fertility and prevention of an ectopic pregnancy that upper genital tract involvement be ruled out. The presentation of a patient with pelvic inflammatory disease (PID) is subtle, in contrast to the presentation of an individual with obvious salpingitis, who has a purulent discharge, cervical motion and adnexal tenderness, fever, and an elevated WBC count. The essence of the examination is the detection of tenderness on motion or palpation of the cervix, uterus, or adnexa. Even though the patient is afebrile with a normal WBC count, she may have vague lower abdominal pain that she does not relate to the physician or is not aware of until a pelvic examination is performed.

**TABLE 3. Fluoroquinolones**

| |
|---|
| Amifloxacin |
| Ciprofloxacin |
| Difloxacin |
| Enoxacin |
| Fleroxacin |
| Lomefloxacin |
| Norfloxacin |
| Ofloxacin |
| Pefloxacin |
| Temafloxacin |
| Tosufloxacin |

### TREATMENT

As mentioned previously, the CDC's recommendation for patients with suspected or proven uncomplicated gonococcal or chlamydial infection is the administration of ceftriaxone, 250 mg IM, followed by doxycycline, 100 gm orally twice daily for 7 days.[14] However, this treatment is costly and time-consuming, and the IM injection may be painful to the patient. The new quinolone ofloxacin offers the opportunity to treat both gonococcal and chlamydial infections with a single oral agent.

The fluoroquinolones are derived from nalidixic acid, which was placed in clinical use in 1962 primarily for the treatment of gram-negative urinary traction infections.[16] The new quinolones differ from nalidixic acid in having a fluorine atom and piperazyl moiety (Table 3). Although the fluoroquinolones all possess the basic quinolone structure—a fluorine atom at the 6 position and a piperazine ring at the 7 position—they differ from one another by substitutions at the 1 position. Ofloxacin is unique among the fluoroquinolones in that it has a tricyclic ring structure. The tricyclic ring results in the molecule's having increased gram-positive and anaerobic activity and decreased metabolism. Ofloxacin has a broad spectrum of activity that includes gram-positive aerobic and facultative anaerobes (Table 4). *Bacteroides fragilis, Bacteroides melaninogenicus, Fusobacterium, Peptostreptococcus,* and *Clostridium difficile* are all sensitive to ofloxacin.[17–20] However, its efficacy in the treatment of

**TABLE 4. Bacterial spectrum of activity of ofloxacin**

| | |
|---|---|
| Enterobacteriaceae | STB[a] |
| Citrobacter | *Hemophilus ducreyi* |
| Enterobacter | *Chlamydia trachomatis* |
| Escherichia | *Neisseria gonorrhoeae* |
| Hafnia | *Ureaplasma urealyticum* |
| Klebsiella | |
| Pseudomonas | |
| Morganella | Gram-positive organisms |
| Proteus | *Staphylococcus aureus*[b] |
| Providencia | *Streptococcus agalactiae* |
| Salmonella | *Streptococcus pneumoniae* |
| Serratia | *Streptococcus pyogenes* |
| Yersinia | *Enterococcus faecalis* |

[a]Sexually transmitted bacteria.
[b]Includes both penicillin-sensitive and penicillin-resistant strains.

infections involving anaerobic bacteria has not been established. Until sufficient data are available, an agent with a proven anaerobic spectrum should be included in the treatment regimen.

The antimicrobial spectrum of activity exhibited by ofloxacin makes it useful for a variety of infections, e.g., skin and soft tissue, respiratory, urinary tract, gonococcal, and chlamydial infections.[21–24] The susceptible minimal inhibitory concentration (MIC) breakpoint is 2 μg/ml, and the MIC for resistance is > 8 μg/ml.[25,26] In vitro and in vivo studies have shown that ofloxacin is as effective as amoxicillin or ampicillin plus probenecid and doxycycline for the treatment of gonococcal and chlamydial infection. In a multicenter study, a single 400-mg dose of ofloxacin was compared with a single 3.0-g dose of amoxicillin or a 4.5-g dose of ampicillin plus 1 g of probenecid. Ofloxacin eradicated the gonococcus in 98% of the patients, whereas the amoxicillin-probenecid regimen eradicated 95%.[24] In a comparative study of the treatment of chlamydia, 533 patients were randomly assigned to a 7-day course of either ofloxacin, 300 mg every 12 hours, or doxycycline, 100 mg every 12 hours. The two agents were reported to be equally effective, achieving cure rates of 99% and 95%, respectively.[27–30]

Although ciprofloxacin has been shown to have activity against *N. gonorrhoeae* and *C. trachomatis*, patients treated for the latter have relapsed. Therefore, while ciprofloxacin appears to be quite adequate for the treatment of *N. gonorrhoeae*, it is not reliable for the treatment of *C. trachomatis*. Norfloxacin also has activity against *N. gonorrhoeae* but not against *C. trachomatis*. The use of norfloxacin to treat cervical infection caused by *N. gonorrhoeae* is appropriate but requires the addition of an agent effective against *C. trachomatis*. To be cost-effective and therapeutically sound, the treatment of cervicitis suspected of being caused by either or both of these sexually transmitted bacteria should be accomplished with a single effective agent such as ofloxacin.

### METABOLISM

Ofloxacin, unlike other fluoroquinolones, is not metabolized to compounds with lesser antimicrobial activity. All fluoroquinolones except ofloxacin are metabolized or produce an oxoquinolone metabolite that interferes with the metabolism of theophylline and caffeine.[31,32] Urinary recovery of metabolites of ofloxacin accounts for approximately 5% of the drug. The dosage of ofloxacin must be adjusted in the face of renal impairment, that is, a creatinine clearance of < 50 ml/min. The half-life of ofloxacin is 9 hours.[33] After the administration of 500 mg of ofloxacin twice a day and achievement of a plasma steady state, the peak level exceeding 5 μg/ml and trough level of 1 μg/ml are obtained.

### TOXICITY

Approximately 3,200 patients in clinical trials in the United States have received ofloxacin for 3 to 10 days. The most frequent adverse reactions were nausea (3.5%), insomnia (1.8%), headache (1.4%), and dizziness (1.2%).

Animal studies revealed that arthropathy, characterized by blisters, erosion, and an increase in synovial fluid, occurred in rats and dogs. The effects were age and dose dependent.[34] These effects are characteristics of all quinolones and, although arthropathy has not been demonstrated in humans, children and pregnant women should not receive fluoroquinolone therapy.

### CONCLUSIONS

Of the quinolones currently available, only ofloxacin presents the physician with the opportunity of treating the two most common sexually transmitted bacterial infections with a single agent. The CDC recommended in 1985 that any treatment for uncomplicated gonorrhea include treatment for chlamydial infection.[14] This recommendation was deemed necessary because of the decrease in efficacy of penicillin in treating gonococcal infection and the increase in the chromosomally mediated resistance of *N. gonorrhoeae* to tetracycline as well as penicillin.[15] Ofloxacin offers the benefits of not being affected by enzymes that afford the gonococcus resistance to tetracycline or penicillins and of being effective as a single dose. The antibacterial activity of the quinolones resides in their ability to interfere with DNA synthesis by inhibiting the bacterial DNA gyrase enzyme that is responsible for the spiral structure producing supercoils, which are characteristic of DNA.[35,36] The fact that only bacteria possess DNA gyrase increases the safety of fluoroquinolones. Since ofloxacin is taken orally and has excellent absorption via the gastrointestinal tract, therapy is facilitated in busy clinical settings in comparison with IM injection of ceftriaxone or 3 to 4.5 g of amoxicillin or ampicillin.

The spectrum of activity of ofloxacin makes this agent potentially suited for the treatment of PID. Initial studies (unpublished) have shown that ofloxacin is as effective as cefoxitin in the treatment of early, uncomplicated PID. The major concern is the decreased activity of quinolones against gram-negative obligate anaerobes. However, an individual experiencing a first episode of PID is primarily infected with *N. gonorrhoeae,* or *C. trachomatis,* or both and is not likely to have a complex infection involving both aerobic and anaerobic bacteria. If there is a concern over a possible mixed aerobic and anaerobic infection, ofloxacin, which provides excellent coverage against *N. gonorrhoeae, C. trachomatis,* Enterobacteriaceae, and gram-positive aerobic cocci, could be administered with an agent such as metronidazole. Additional studies are needed to determine if ofloxacin is indicated in the treatment of PID.

There is no doubt that ofloxacin is well suited for the treatment of uncomplicated gonococcal and chlamydial infection. Although ofloxacin has been associated with low toxicity, arthropathic effects have been demonstrated in rats and dogs. Therefore, ofloxacin, like other quinolones, should not be administered to children younger than 18 years of age or to pregnant or breastfeeding women.

### REFERENCES

1. Faro S: *Chlamydia trachomatis* infection in women. J Reprod Med 30:273–278, 1985.
2. McCormack WM, Rosner B, McComb DE, et al.: Infection with *Chlamydia trachomatis* in female college students. Am J Epidemiol 121:107–115, 1985.
3. Paavonen J, Roberts PL, Stevens CE, et al.: Randomized treatment of mucopurulent cervicitis with doxycycline or amoxicillin. Am J Obstet Gynecol 161:128–135, 1989.
4. Phillips RS, Safran C, Aronson MD, Taylor WC: Should women be tested for gonococcal infection of the cervix during routine gynecologic visits? An economic appraisal. Am J Med 86:297–302, 1989.
5. Backman M, Ruden A-K, Bygdeman SM, et al.: Gonococcal serovar distribution in Stockholm with special reference to multiple infections and infected partners. Acta Pathol Microbiol Immunol Scand Sect B 93:225–232, 1985.
6. Covino JM, Cummings M, Smith B, et al.: Comparison of ofloxacin and ceftriaxone in the treatment of uncomplicated gonorrhea caused by penicillinase-producing and non-penicillinase-producing strains. Antimicrob Agents Chemother 34:148–149, 1990.
7. Kirshon B, Faro S, Phillips LE, Pruett K: Correlation of ultrasonography and bacteriology of the endocervix and posterior cul-de-sac of patients with severe pelvic inflammatory disease. Sex Transm Dis 15:103–107, 1988.
8. Washington AE, Cates W, Zaidi AA: Hospitalization for pelvic inflammatory disease. JAMA 25:2529–2533, 1984.
9. Chaim W, Sarov B, Sarov I, Piura B, Cohen A, Insler V: Serum IgG and IgA antibodies to chlamydia in ectopic pregnancies. Contraception 40:59–71, 1989.
10. Conway D, Glazener CM, Caul EO, et al.: Chlamydial serology in fertile and infertile women. Lancet 1:191–193, 1984.
11. Dodson MG, Faro S, Gentry LO: Treatment of acute pelvic inflammatory disease with aztreonam, a new monocyclic beta-lactam antibiotic, and clindamycin. Obstet Gynecol 67:657–662, 1986.
12. Ginsberg K, Faro S: Management of pelvic inflammatory disease. Infect Surg 6:562–567, 1987.
13. Svensson L, Mardh PA, Westrom L: Infertility after acute salpingitis with special reference to *Chlamydia trachomatis*. Fertil Steril 40:322–329, 1983.
14. Centers for Disease Control: 1985 STD treatment guidelines. MMWR 34(Suppl 45):755–1085, 1985.
15. Rice RJ, Thompson SE: Treatment of uncomplicated infections due to *Neisseria gonorrhoeae.* A review of clinical efficacy and in vitro susceptibility studies from 1982 through 1985. JAMA 255:1739–1746, 1986.
16. Lesher GY, Froelich ED, Gruet MD, et al: 1,8 Naphthyridine derivatives. A new class of chemotherapeutic agents. J Med Pharm Chem 5:1063, 1962.
17. Gruneberg RN, Felmingham D, O'Hare MD, et al.: The comparative in vitro activity of ofloxacin. J Antimicrob Chemother 22(Suppl C):9–19, 1988.
18. van Caekenberghe DL, Pattyn SR: In vitro activity of ciprofloxacin compared with those of new fluorinated piperazinyl-substituted quinolone derivatives. Antimicrob Agents Chemother 25:518–521, 1984.
19. King A, Phillips I: The comparative in vitro activity of eight newer quinolones and nalidixic acid. J Antimicrob Chemother 18(Suppl D):1–20, 1986.

20. Delmee M, Avesani V: Comparative in vitro activity of seven quinolones against 100 isolates of *Clostridium difficile*. Antimicrob Agents Chemother 29:374–375, 1986.
21. Gentry LO, Rodriguez-Gomez G, Zeluff BJ, Khoshdel A, Price M: A comparative evaluation of oral ofloxacin versus intravenous cefotaxime therapy for serious skin and skin structure infections. Am J Med 87(Suppl 6C): 57S–60S, 1989.
22. Cox CE: Ofloxacin in the management of complicated urinary tract infections, including prostatitis. Am J Med 87(Suppl 6C):61S–68S, 1989.
23. Stocks JM, Wallace RJ, Griffith DE, Garcia JG, Kohler RB: Ofloxacin in community-acquired lower respiratory infections. A comparison with amoxicillin or erythromycin. Am J Med 87(Suppl 6C):52S–56S, 1989.
24. Lutz FB: Single-dose efficacy of ofloxacin in uncomplicated gonorrhea. Am J Med 87(Suppl 6C):69S–74S, 1989.
25. Fuchs PC, Barry AL, Jones RN, Thornsberry C: Proposed disk diffusion susceptibility criteria for ofloxacin. J Clin Microbiol 22:310–311, 1985.
26. National Committee for Clinical Laboratory Standards: Performance Standards for Antimicrobial Susceptibility Testing; Second Informational Supplement. Villanova, PA: NCCLS, M100-S2, 1987.
27. Schachter J, Moncada JV: In vitro activity of ofloxacin against *Chlamydia trachomatis*. Am J Med 87(Suppl 6C): 14S–16S, 1989.
28. Batteiger BE, Jones RB, White A: Efficacy and safety of ofloxacin in the treatment of nongonococcal sexually transmitted disease. Am J Med 87(Suppl 6C):75S–77S, 1989.
29. Judson FN, Beals BS, Tack KJ: Clinical experience with ofloxacin in sexually transmitted disease. Infection 14(Suppl 4):S309–S310, 1986.
30. Fransen L, Avonts D, Piot P: Treatment of genital chlamydial infection with ofloxacin. Infection 14(Suppl 4):S318–S320, 1986.
31. Edwards DJ, Bowles SK, Svensson CK, Rybak MJ: Inhibition of drug metabolism of quinolone antibiotics. Clin Pharmacokinet 15:194–204, 1988.
32. Wijnands WJ, Vree TB, van Herwaarden CL: The influence of quinolone derivatives on theophylline clearance. Br J Clin Pharmacol 22:677–683, 1986.
33. Flor S: Pharmacokinetics of ofloxacin: An overview. Am J Med 87(Suppl 6C):24S–30S, 1988.
34. Davis GJ, McKenzie BE: Toxicologic evaluation of ofloxacin. Am J Med 87(Suppl 6C):43S–46S, 1989.
35. Gellert M, Mizuuchi K, O'Dea MH, Nash HA: DNA gyrase: An enzyme that introduces superhelical turns into DNA. Proc Natl Acad Sci USA 73:3872–3876, 1976.
36. Cook TM, Brown KG, Guss WA: Bactericidal action of nalidixic acid on *B. subtilis*. J Bacteriol 92:1510–1514, 1966.
